# Dopamine-2 receptor antibody encephalitis presenting as pure tongue-biting in a tourette syndrome patient: a case report

**DOI:** 10.1186/s12888-021-03683-4

**Published:** 2022-01-20

**Authors:** Mingfeng Lai, Yuanyuan Li, Dan Luo, Jiajun Xu, Jing Li

**Affiliations:** Mental Health Center West China Hospital, Sichuan University, No. 28 Dian Xin Nan Road, Sichuan, Chengdu 610041 China

**Keywords:** D2R encephalitis, Tourette syndrome, Premonitory urges, Immunotherapy, D2R antagonists

## Abstract

**Background:**

Tourette syndrome (TS) is a neuropsychiatric disorder characterized by repetitive and patterned tics. Its onset correlates with dysfunctions in immunological activation and neurotransmitters. Autoimmune movement disorders such as dopamine-2 receptor antibody encephalitis (D2R encephalitis) may go undiagnosed in TS patients seeking medical help for tic symptoms only. Here, we present a clinical case of D2R encephalitis in a TS patient.

**Case presentation:**

A 13-year-old boy with a history of TS presented with acute tongue-biting without positive neurologic examination or auxiliary examination results, except for a weakly positive finding for D2R antibodies in the serum sample. He was initially diagnosed with possible D2R encephalitis, but the influence of TS could not be ruled out. In addition to psychotropics, we administered immunotherapy early based on clinical characteristics, and his symptoms were ameliorated significantly. During the follow-up, he was diagnosed with definite D2R encephalitis, and the dosage of psychotropics was further adjusted for fluctuating symptoms.

**Conclusions:**

Our case suggests that clinicians should discern D2R encephalitis in TS patients when tics are the primary symptoms. Administering immunotherapy early, according to clinical characteristics, may benefit the patient. Moreover, the features of premonitory urges could help evaluate the state of TS.

## Background

Dopamine-2 receptor antibody encephalitis (D2R encephalitis), usually known as basal ganglia encephalitis, has been acknowledged as a rare subtype of autoimmune encephalitis since the discovery of antibodies targeting the N-terminal of D2R on the neuronal surface [1]. The general prevalence and incidence rates of autoimmune encephalitis at 13.7/100,000 and 0.8/100,000 person-years, respectively, are comparable to those of infectious encephalitis, but it has a higher rate of relapse and poorer prognosis [2, 3]. The epidemiological and prognostic data of D2R encephalitis remain unclear. Its main encephalopathy syndrome is prominent movement disorder (such as dystonia, chorea, or tics) while main psychiatric features are agitation, psychosis, and emotional instability [1, 4, 5]. Negative results of magnetic resonance imaging (MRI), cerebrospinal fluid (CSF), or neurologic examinations in nearly half of the patients [4, 6] and non-specific tics or psychiatric performances result in D2R encephalitis patients with a history of Tourette syndrome (TS) being misdiagnosed in psychiatric clinics. According to the Diagnostic and Statistical Manual of Mental Disorders, fifth edition (DSM-V) classification, TS is a childhood-onset disorder characterized by motor and verbal tics, which were present at some time during the illness concurrently or separately and persisted for over a year. The prevalence of TS in children is reported to be 0.52–0.77% [7, 8], and the lifetime prevalence of any psychiatric comorbidity with TS is 85.7% [9]. Here we present a case of D2R encephalitis presenting as pure tongue biting in a TS patient.

## Case presentation

A Chinese boy developed multiple motor tics, such as eye blinking, shoulder elevation, throat clearing, and vocal tics, such as saying “I want to rape you” when he was 9 years old in 2016. He also had a history of repeatedly and uncontrollably examining doors or windows and was diagnosed with TS and obsessive–compulsive disorder (OCD). The above symptoms disappeared after he was prescribed 50 mg/d sertraline, 10 mg/d aripiprazole and 1 mg/d risperidone. In June 2020, he developed frequent tongue-biting, which manifested as a sudden, rapid, recurrent, non-rhythmic, aimless, involuntary behaviour after getting oral ulcers. The symptom slightly abated after the ulcer healed but aggravated once more when he caught a cold. The patient appeared agitated and bit his tongue frequently when he presented to our inpatient ward in July 2020. He presented with continuous oral bleeding and a necrotic anterior third of the tongue with no symptoms of vomiting, diarrhoea, arthritis or cardiac changes and negative neurologic examination results. A third generation cephalosporin was not effective in preventing the tongue-biting. We administered 20 mg/d haloperidol and 20 mg/d diazepam to prevent further injury of the tongue, which was still severely injured.

We arranged an emergency operation to remove the necrotic tongue tissue; we used gauze instead of hard mouth openers, administered 6 mg/d trihexyphenidyl to prevent extrapyramidal reactions, and performed lumber puncture to rule out intracranial infection. After his subjective discomfort reduced, both tongue-biting and agitation subsided to some extent, and the patient became capable of describing important sensory experiences on day 3 (three days after the first admission). The tongue-biting seemed to have different features: sometimes it was unanticipated and severely injurious, often accompanied by bleeding and only temporarily relieved by sedative drugs. Other times, it was premonitory and temporary. When the patient felt pressure, tension, entanglement, or discomfort, he would utter a premonition of “I am going to bite, it is coming." He could not completely control the tongue-biting but stopped automatically after obtaining satisfaction and comfort. Compulsive and autistic characteristics were also observed: a feeling of “not just right” appeared when his mobile phone was not fully charged, or a bunch of grapes were not fully eaten; he felt immense discomfort and needed to repeat tongue-biting from the beginning when the process was interrupted. He always wore black clothing in the same style, spoke in a flat tone, and asked others to respond with specific words in a particular posture. There was no history of growth retardation, encephalitis, or poor concentration level compared with that of his peers.

**Fig. 1:**
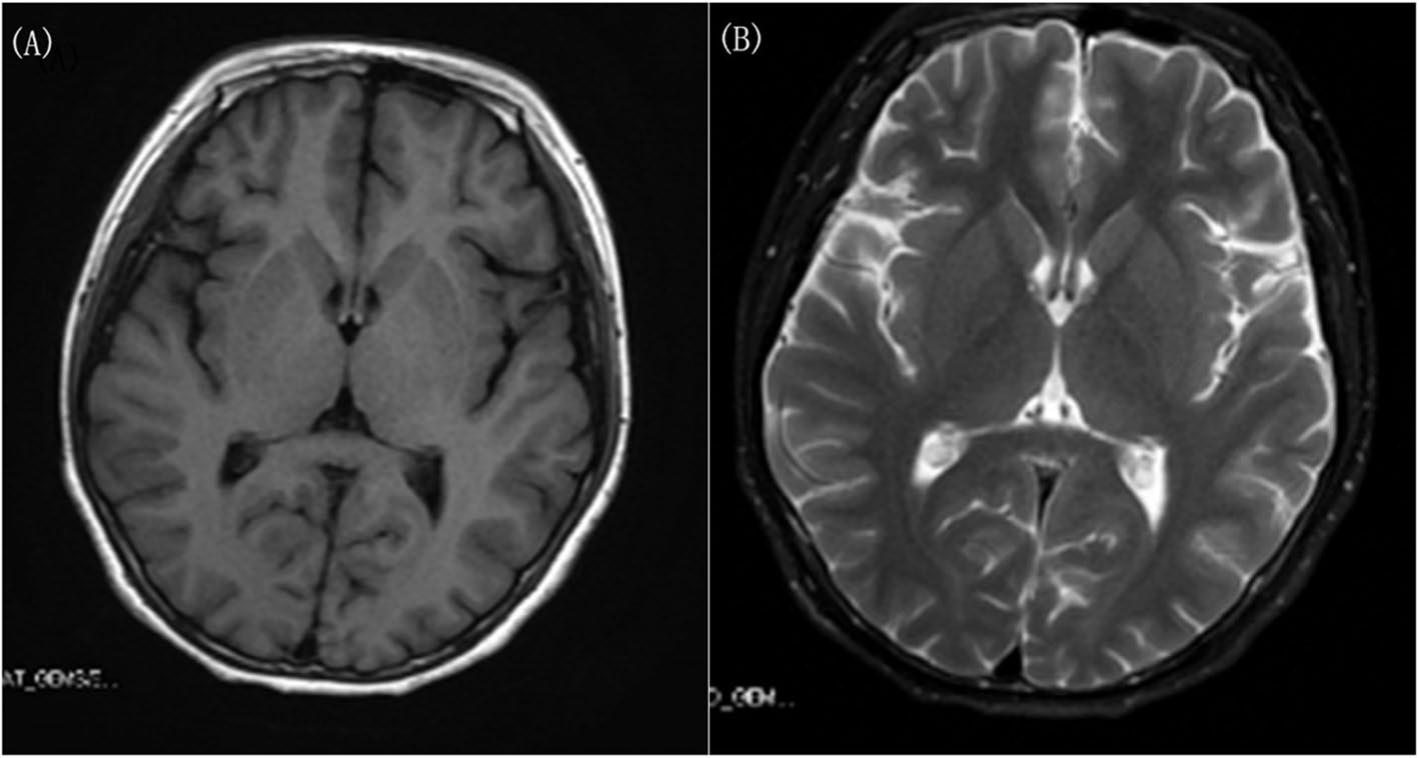
Brain magnetic resonance imaging in July 2020. (**a)** No positive results were found in the T1 sequence (**b)** no positive results were found in the T2 sequence

The laboratory results concerning blood cell count, red blood cell morphology, liver and kidney function, electrolytes, blood lipids, lactic acid, ceruloplasmin, anti-streptolysin-O (ASO), CSF protein level and white cell count, TORCH pathogens (*Toxoplasma gondii*, *rubella* virus, *cytomegalovirus*, and *herpes simplex* virus) and findings of electroencephalogram (EEG), electrocardiogram, and brain MRI **(Figs. ****1****a and b)** were all normal.

**Fig. 2:**
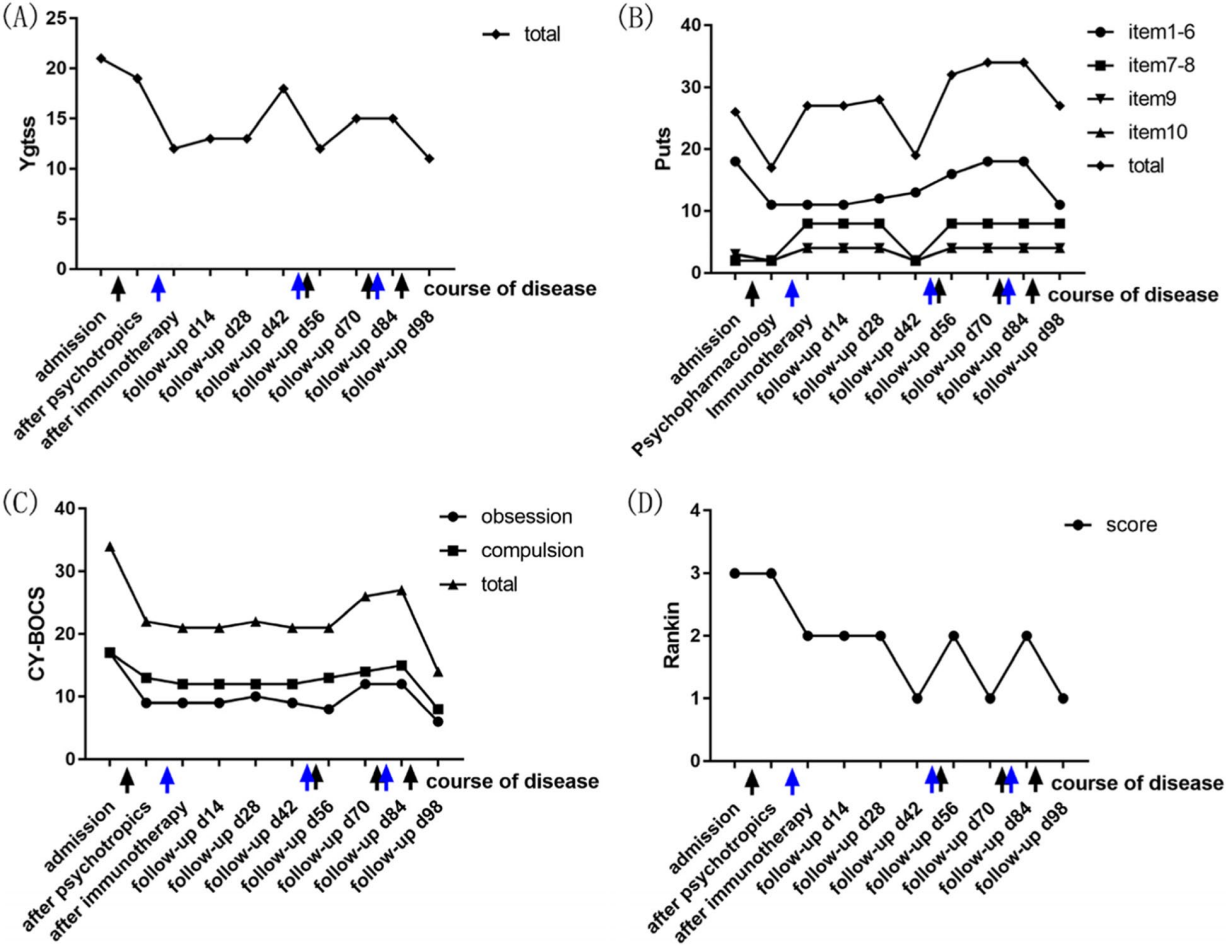
Clinical assessment of the symptoms. The time course of YGTSS, PUTS, CY-BOCS, mRS scores are depicted on (**a**), (**b**), (**c**), and (**d**) subplots, respectively. The patient was independently scored at 10 time points throughout the treatment and follow-up course (x axis) by the two first authors of the paper, and a mean score was obtained. Major interventions are marked below the x axis. The black arrows represent the four adjustments to the patient’s psychotropic regimen: 1, in addition to sertraline 200 mg/d, the patient was first prescribed risperidone 6 mg/d for 5 days, then risperidone 6 mg/d and olanzapine 10 mg/d for 5 days, and finally risperidone 6 mg/d and aripiprazole 10 mg/d for 2 days; 2, sertraline, risperidone and aripiprazole were discontinued; 3, risperidone 6 mg/d, aripiprazole 10 mg/d and sertraline 50 mg/d were re-administered for 2 weeks; and 4, aripiprazole was adjusted to 20 mg/d, fluvoxamine was prescribed at 200 mg/d, and lorazepam was prescribed at 1.5 mg/d. The blue arrows represent the three rounds of immunotherapy: 1, methylprednisolone 1000 mg/d for 5 days; 2, methylprednisolone 1000 mg/d for 5 days; and 3, immunoglobulin 22.5 g/d for 5 days and methylprednisolone 1000 mg/d for 3 days. (**a**) YGTSS score indicates tic severity by number, frequency, complexity, interference, and impairment. (**b**) PUTS: Scores for items 1–6 indicate intensity of PU; items 7–8 indicate frequency of PU; items 9–10 indicate the ability of voluntary suppression. (**c**) CY-BOCS score indicates the severity of obsession and compulsion. (**D**) mRS score indicates neurological function. YGTSS, Yale Global Tic Severity Scale; PUTS, Premonitory Urge for Tics Scale, CY-BOCS, Children's Yale-Brown Obsessive–Compulsive Scale; mRS, modified Rankin Scale; PU, premonitory urge

**Fig. 3:**
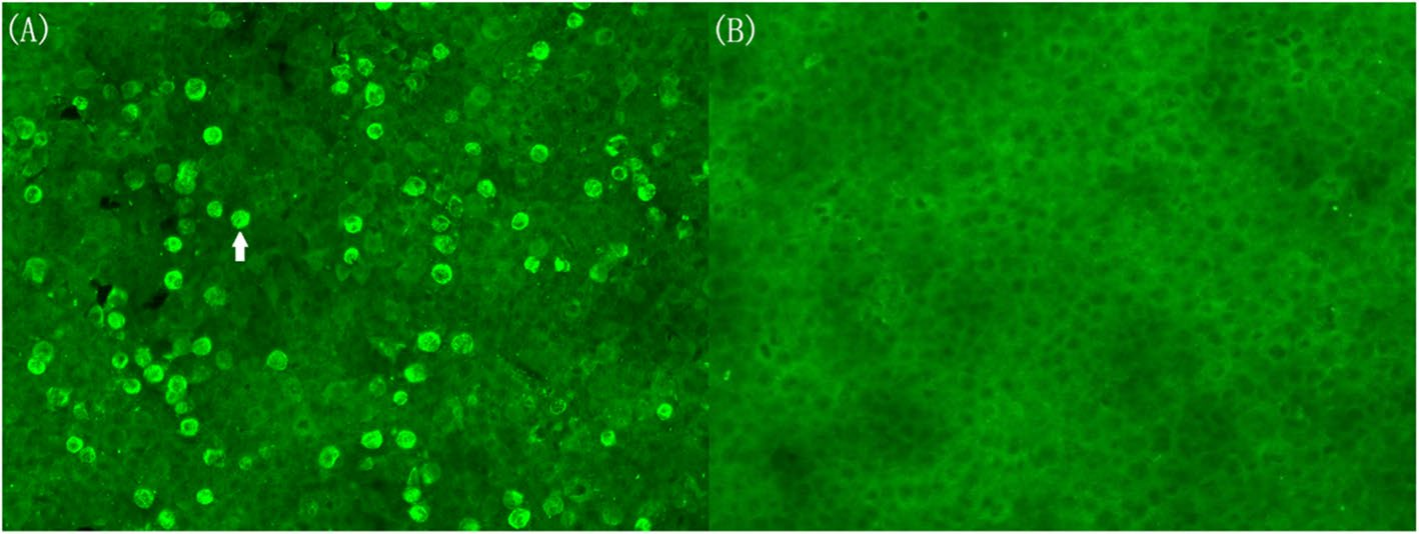
Serum sample with anti-D2R antibody detected using cell-based assays in July 2020. **(a)** The arrow points to one positive cell diluted in the ratio of 1:32. (**b)** The negative results from the control group

Before the results of CSF antibodies tests came out, we treated his tongue-biting behaviour as a new manifestation of TS; we employed the Yale Global Tic Severity Scale to assess the severity of tic symptoms [10], the Premonitory Urge for Tics Scale to assess the characteristics of premonitory urges (PU) [11], the Children's Yale-Brown Obsessive–Compulsive Scale to assess obsessive–compulsive symptoms [12], and the modified Rankin Scale to assess neurological function [13] **(Figs. ****2****a–d)**. The patient scored 42 and 4 in the Autistic Behavior Checklist (ABC) [14] and Swanson, Nolan, and Pelham Parent Rating Scale (SNAP-IV) [15] respectively, which were unable to diagnose autism spectrum disorder and attention deficit hyperactivity disorder. His attention level was better than 5–15% of the population in the Integrated Visual and Auditory Continuous Performance Test (IVA-CPT). His intelligence quotient (IQ) score was 85, better than 20% of the population in the Raven’s Standard Progressive Matrices (SPM). He scored 87–93, lower than the average level in the visual and verbal memory subscales of Children’s Memory Scale (CMS). He was prescribed 6 mg/d risperidone for 5 days, which was adjusted to 6 mg/d risperidone and 10 mg/d olanzapine for the following 5 days, and finally to 6 mg/d risperidone and 10 mg/d aripiprazole due to insensitivity to D2R antagonists. We concurrently prescribed 200 mg/d sertraline to improve the patient’s obsessive–compulsive symptoms. However, unanticipated tongue-biting with bleeding was still frequent on day 12. TS patients with refractory tics and severe self-injury can be treated with deep brain stimulation [16], but the patient’s age precludes this option. On day 12, the D2R antibody titre detected by cell-based assays in the serum sample showed a ratio of 1:32, while the CSF sample showed negative findings **(Figs. ****3****a and b)**. The other 15 different autoimmune encephalitis antibodies, including anti-NMDAR and anti-CASPR, paraneoplastic antibodies and oligoclonal bands in both samples were negative. After consultation with senior neurologists, the patient was diagnosed with possible D2R encephalitis based on the clinical features of acute onset movement disorders and D2R antagonist insensitivity. With parental consent, he was administered immunotherapy (1000 mg/d methylprednisolone for 5 days) to control the tongue-biting as soon as possible. Consequently, the frequency of tongue-biting decreased significantly by day 20, and he was administered 50 mg/d prednisone to continue immunotherapy after discharge.

**Fig. 4:**
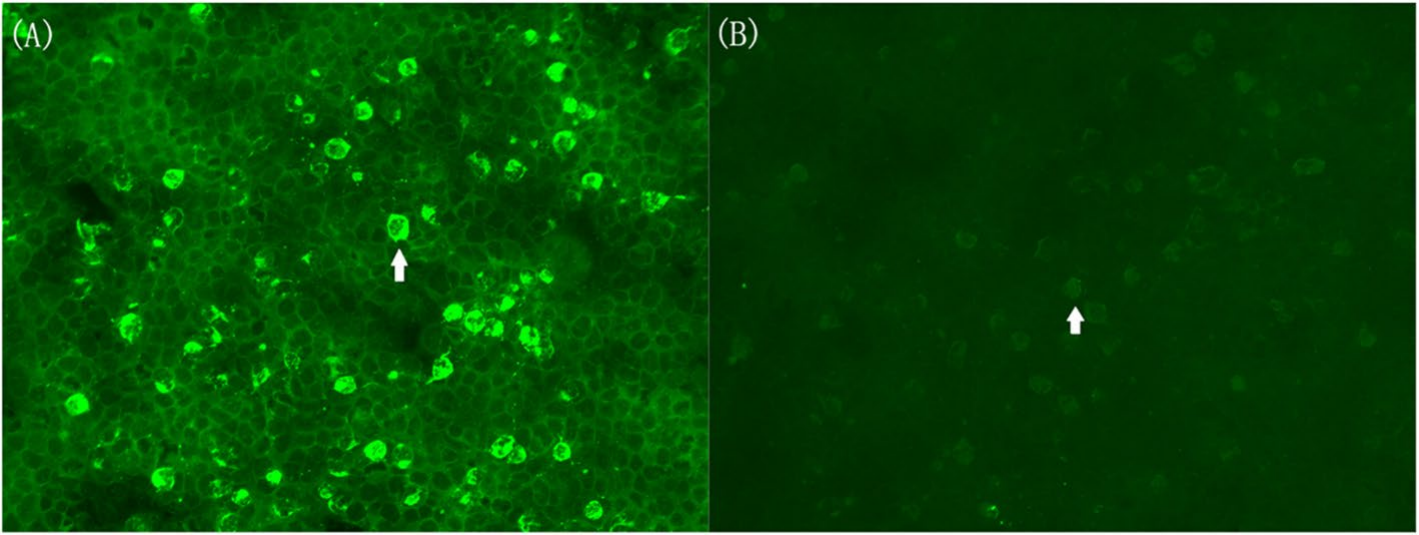
Serum and CSF samples with anti-D2R antibody detected using cell-based assays in September 2020. **(a)** The arrow points to one positive cell diluted in the ratio of 1:100 in serum sample. (**b)** The arrow points to one positive cell diluted in the ratio of 1:1 in CSF sample. CSF, cerebrospinal fluid

The patient was followed up by telephone or face-to-face meetings every two weeks after discharge **(Figs. ****2****a–d)**. It is worth mentioning that there were two incidences of recurrence with different clinical features. Unanticipated tongue-biting and bleeding reoccurred on follow-up day 42 (62 days after the first admission) when the dosage of prednisone was reduced to 30–40 mg/d. The frequency of PU (**Fig. ****2****b, items 7–8**) and ability of voluntary suppression (**Fig. ****2****b, items 9–10**) decreased sharply. The follow-up D2R antibody titres in the serum and CSF sample were 1:100 and 1:1, respectively **(Fig. ****4**** a and b)**. MRI and ASO were also normal, and video EEG lacked evidence to support a diagnosis of epilepsy. Definite D2R encephalitis was then diagnosed, and tongue-biting decreased after another round of immunotherapy (1000 mg/d methylprednisolone for 5 days) by follow-up day 56. However, premonitory tongue-biting became frequent with the intensity of PU (**Fig. ****2****b, items 1–6**) and obsessive–compulsive symptoms **(Fig. ****2****c)** increasing significantly on follow-up day 70. During this period, sertraline, risperidone, and aripiprazole were discontinued for more than 2 weeks. The tongue-biting did not improve much after these drugs were re-administered. D2R antibody titre (in serum and CSF), MRI, ASO, and video EEG were re-examined and yielded negative results. The improvement in tongue-biting was unsatisfactory at follow-up day 84 after immunotherapy (22.5 g/d immunoglobulin for 5 days and 1000 mg/d methylprednisolone for 3 days). Considering the characteristic PU and compulsive symptoms, the psychotropics were adjusted to 20 mg/d aripiprazole, 200 mg/d fluvoxamine, and 1.5 mg/d lorazepam (dosage gradually increased within a week). Although we cannot completely rule out effects of immunotherapy after discharge, the remission of tongue-biting was closely related to the increased dosage of psychotropics according to the parents’ observation at follow-up day 98. The cognitive function also recovered at the ending of follow-up, with the attention level better than 45–55% of the population, IQ score elevating to 92 and memory scores elevating to 98–103.

The patient was satisfied with the treatment that he had received and felt an improvement in his symptoms. His quality of life was almost unaffected by his symptoms at follow-up day 98. The patient and his parents have stated that they will not reduce or discontinue the psychotropics again without the psychiatrist’s approval—even if the tics become stable or fluctuate over a short period.

## Discussion and conclusions

To our knowledge, this is the first clinical case report of D2R encephalitis presenting as tongue-biting in a TS patient without characteristic neurological signs or positive auxiliary examination results. In our case, acute exacerbation of tongue-biting and insensitivity to D2R antagonists, the first-line medication for TS, made us consider the possibility of a neurological disease. There were no imaging features of space occupying lesion, focal vessel lesion, typical demyelination or substance deposition near the basal ganglia in brain MRI. The negative results in the blood and CSF sample were also insufficient to diagnose vasculitis, inflammatory, demyelinating or metabolic diseases. It needed to mention that the patient did not meet the criteria of paediatric autoimmune neuropsychiatric disorders associated with streptococcal infections or Sydenham’s chorea, considering no benefit from anti-streptococcal therapy and multiple negative ASO results. Epilepsy and acanthocytosis were also preliminarily excluded, based on the negative results of video EEG and red blood cell morphology. Thus, the positive finding in D2R antibody was an important clue. Although some researchers doubt the clinical significance of antibodies only in serum considering the possibility of false positive results [17], others believe false negative results for antibody titre in CSF may occur due to immunoprecipitation of the brain tissue as well [18]. Overall, antibody detection at different institutions may be prone to detection errors, but the antibody status does not appear to influence the effect of immunotherapy [19]. Therefore, we administered immunotherapy at an early stage based more on clinical characteristics than on antibody results, especially of the first and third CSF examination. Our approach was based on the recommendations of a study that stated clinical decisions should be based on clinical assessment when regular follow-up and baseline determination of serum and CSF titres after recovery cannot be obtained [20]. We were also suspicious whether an autoimmune reaction was triggered by tumour molecular mimicry and tested for 13 different paraneoplastic antibodies, including anti-Hu and anti-Yo antibodies, which yielded negative results. These negative results are consistent with the conclusion of a previous study that reported the frequency of cancer in D2R encephalitis as 0% [1]. Fluorodeoxyglucose positron emission tomography/computed tomography is more sensitive than computed tomography/MRI for detecting autoimmune encephalitis and tumours [21] but was refused by the patient’s parents for economic reasons.

On the other hand, an influence of TS on the patient’s tongue-biting could not be ruled out, and four factors needed to be considered. First, multiple and typical motor tics and vocal tics were observed during the year long course of the disease, although not concurrently. There was sufficient basis for a diagnosis of TS according to DSM-V. Second, the tongue-biting behaviour also showed sudden, rapid, recurrent, non-rhythmic, aimless, involuntary characteristics, which fully fitted the definition of tics. Tic symptoms usually fluctuate and get aggravated by some triggers, often after a long period of remission. The development of oral ulcers and a cold could have been these triggers. Third, premonitory feelings, compulsions, and autistic features that accompanied the tongue-biting behaviour are common psychological factors in TS patients. Previous studies found that 72.1% of TS patients meet the criteria for OCD or attention-deficit/hyperactivity disorder [9] and 4.6–18% meet the cut-off criteria for autism spectrum disorder [22, 23]. Up to 93% of TS patients report PU, a characteristic uncomfortable experience [24]. They usually feel itchy, pressure, and tense inside [25], and some report a feeling of “something is not complete” or “not just right.” Fourth, the adjustment of psychotropics resulted in improvement in tongue-biting to some extent, partly by decreasing the intensity of PU and compulsions. Fluvoxamine and aripiprazole are seemed to be potential treatment options. Fluvoxamine is believed to be efficacious in treating OCD in children [26], while aripiprazole is a unique D2R antagonist with multiple mechanisms that modulate not only the dopaminergic neurotransmitter release but also glutamatergic and GABAergic neurotransmitter systems [27]. Focalized ratios of glutamate and gamma-aminobutyric acid have been shown to influence dopaminergic signalling and cortical outputs, which result in movement disorders [28]. Conversely, drug withdrawal has a negative impact on tongue-biting during follow-up and even intensive immunotherapy cannot achieve complete relief of symptoms.

Reviewing the previous literature, we found some clues to explain the insensitivity to psychotropics and similar symptoms between D2R encephalitis and TS. In an in vitro experiment, D2R antibodies were discovered to correlate with the internalization of D2R [1]. Under this condition, D2R detaches from the cytomembrane and enters the cytoplasm by endocytosis. The indirect dopamine signalling pathway (inhibitory effect) modulated by D2R is blocked, while the direct pathway (excitatory effect) modulated by D1R is activated due to relatively increased D1R binding. Decreased number of D2R antagonist targets may be the cause of psychotropic insensitivity. However, whether the same mechanism is responsible in vivo needs further experiments. For the similarity in symptoms between D2R encephalitis and TS, we hypothesize that basal ganglia changes and dopamine metabolic disorder, the core features of D2R encephalitis, perhaps exist in TS patient as well. Activation in the striatum was found in TS patients [29], while different conclusions regarding volume changes of the putamen and caudate nucleus exist due to comorbidity with OCD or attention-deficit/hyperactivity disorder, severity of tics, age of individuals, and single nucleotide polymorphisms [30–34]. Additionally, increased binding of the dopamine transporter in the striatum [35, 36] and elevated methylation level of the *DRD2* gene [37] were found in TS patients; however, the increased binding of vesicular monoamine transporter type 2 and decreased availability of D2R are still controversial findings [36, 38–40]. Moreover, a hyperactive immune response may be involved in the pathogenesis of TS. Approximately 36% of individuals with TS/chronic tic disorders have autoimmune diseases as comorbidities [41]. Moreover, increased activation of striatal microglia and existence of pathological oligoclonal bands in CSF are discovered [42, 43]. However, in our case, the guardian’s refusal limited further investigations on neuroimaging and metabolomics, which should be explored in future high-quality studies.

For TS patients primarily presenting with tics, especially with acute exacerbation of tics, insensitivity to D2R antagonists, and lack of clinical features such as PU, clinicians should pay attention to the possibility of D2R encephalitis. We suggest administering immunotherapy early when D2R encephalitis is suspected and monitoring antibody levels during the treatment. Employing tools such as the Premonitory Urge for Tics Scale can help evaluate the state of TS. However, further high-quality studies are required to promote development in this field.

## Data Availability

Data sharing is not applicable to this article as no datasets were generated or analyzed during the current study.

## Abbreviations

ASO: Anti-streptolysin-O
CSF: Cerebrospinal fluid
D2R: Encephalitis, dopamine-2 receptor antibody encephalitis
EEG: Electroencephalogram
MRI: Magnetic resonance imaging
OCD: Obsessive–compulsive disorder
PU: Premonitory urges
TS: Tourette syndrome
